# Stabilising proteins in solution using affordable and readily available small molecules

**DOI:** 10.1007/s12551-025-01341-7

**Published:** 2025-08-05

**Authors:** Sandrine Bakam Tchiakam, Sarah L. Berger, June Southall, Helen Walden, Mads Gabrielsen

**Affiliations:** 1 School of Molecular Biosciences, University of Glasgow, Glasgow, G12 8QQ UK; 2 Neil Bulleid Integrated Protein Analysis Facility, MVLS Shared Research Facilities, University of Glasgow, Glasgow, G12 8QQ UK

**Keywords:** Protein purification, Protein stability, Small molecule additives

## Abstract

Purified proteins are sitting in a mostly aqueous environment, with normally some buffer and salt making up the conditions. This is vastly different from their natural habitat, and protein are often affected by this difference, showing signs of destabilisation or aggregation. A common method to improve the protein solubility and homogeneity is adding small molecules to the buffer conditions, as these can aid protein stability and keep the protein in solution at a concentration which is within that needed for the experiments that are to be undertaken. This review is detailing some of the small molecules that are routinely used, with a focus on them being readily available and affordable for all labs. Some of the more common small molecule additives described in this paper are (1) amino acids, like arginine or glycine, (2) sugars, like sucrose, or (3) other osmolytes, such as glycerol. The second part is covering some of the methods that can be utilised to determine whether a small molecule improves the stability of a particular protein.

## Introduction

This review is focused on methods to stabilise a protein in solution, using small molecules. As the authors work at a core research facility within a university, producing proteins or supervising students who are using the facilities to express and purify their own proteins, the focus has been on low-cost compounds that should be readily available to any lab, rather than pursuing more specialised compounds which may require bespoke synthesis or which would be a more substantial investment both in time and money. Also included is a brief description of measurements that can be used to quantify the impact small molecules may or may not have on the protein sample. Again, the methods described are relatively fast and requires small amount of sample. They are routinely used in laboratories, and most researchers should be able to arrange access.

A protein’s structure is strongly correlated to its function. Defined by the amino acid sequence, the stability of the secondary, tertiary, and quaternary structures of proteins are based on weak noncovalent interactions; hydrogen bonding, electrostatic attractions, van der Waals forces, and hydrophobic interactions. The structure of a protein not only determines its binding partners and, consequently, the downstream pathways that it can activate or inactivate but also dictates how it can be modified, for instance, by acetylation, phosphorylation, or ubiquitination.

Proteins in their natural habitat, in vivo or *in cell*, are sitting in a crowded environment consisting of a huge array of other proteins, small molecules, salt, carbohydrates, and lipids. However, to determine the structure of a protein, characterise it biophysically or biochemically, or study its’ mechanism in detail, it needs to be purified and maintained in a fundamentally aqueous environment. This environment often has some buffering capacity (usually Tris, HEPES, or Phosphate in the range of pH 6–8), with some salt present (often NaCl or KCl in a concentration range between 50 and 250 mM), but far less components than what would be found in its natural environment. It should therefore not be surprising if the protein is not in a perfect, homogenous, well-ordered state, and this may lead to a lack of stability where the protein becomes disordered, leading to loss-of-function, aggregation, or precipitation. Correct protein folding involves the buffer entropy gain from the burial of hydrophobic groups, and the enthalpy gain of polar and van der Waals interactions. The native state of a protein is associated with its conformational biological function, whereas the denatured state tends to be associated to aggregation. Stability of a protein is the balancing result between destabilising and stabilising forces. Most proteins are generally unstable, and changes in the protein environment due to differences in the chemical buffer composition, pH, ionic strength, and temperature gradient may result in a destabilising event and alter the native shape. The overall stability of a protein can be measured by the difference between the entropic and enthalpic energy of the native state and the denatured state. The Gibbs free energy, ΔG, of unfolding is a measure of protein stability. When the stabilizing contributions and the destabilizing contributions are summed, and the net result is a negative ΔG, the protein favours a folded conformation (Pace et al. 2014). The melting temperature of a protein (T_m_) can be used to measure stability of a protein. At the T_m_, the ΔG of the unfolded and the folded state are equal, and the T_m_ defines the melting point where half the amount of protein is folded and half unfolded. Other parameters to consider are T_onset_, the temperature at the beginning of the unfolding event, and T_agg_, the temperature at the beginning of protein aggregation. When the ΔG is paired with the T_m_ the ΔH, the enthalpy of unfolding, and the ΔS, the entropy of unfolding, can be extrapolated (Kuril 2024). The stability of a protein or protein complex in solution is the one key parameter that determines whether a biochemical assay or a biophysical experiment has any chance of succeeding. If structural determination techniques such as crystallography, cryo-EM or NMR are the aim of the study, the success-rate will be dramatically reduced by the protein not being in a state conducive to crystallisation or amenable to freezing on cryoEM grids. Likewise, correctly determining any binding affinities between the unstable sample and other binding partners will also be difficult, as it will not be clear what the true, or active, concentration of the sample is (Rahban et al. 2023). Additionally, a disordered sample may expose hydrophobic areas which are not normally available to binding and give false positives or unravel or occlude binding sites that are there. Any biochemical assays will be hampered by the same uncertainty regarding the active concentration, increased noise contribution and reduced signal-to-noise ratio, and by the occlusion of sites involved in the reaction assessed by the undertaken experiment.

**Fig. 1:**
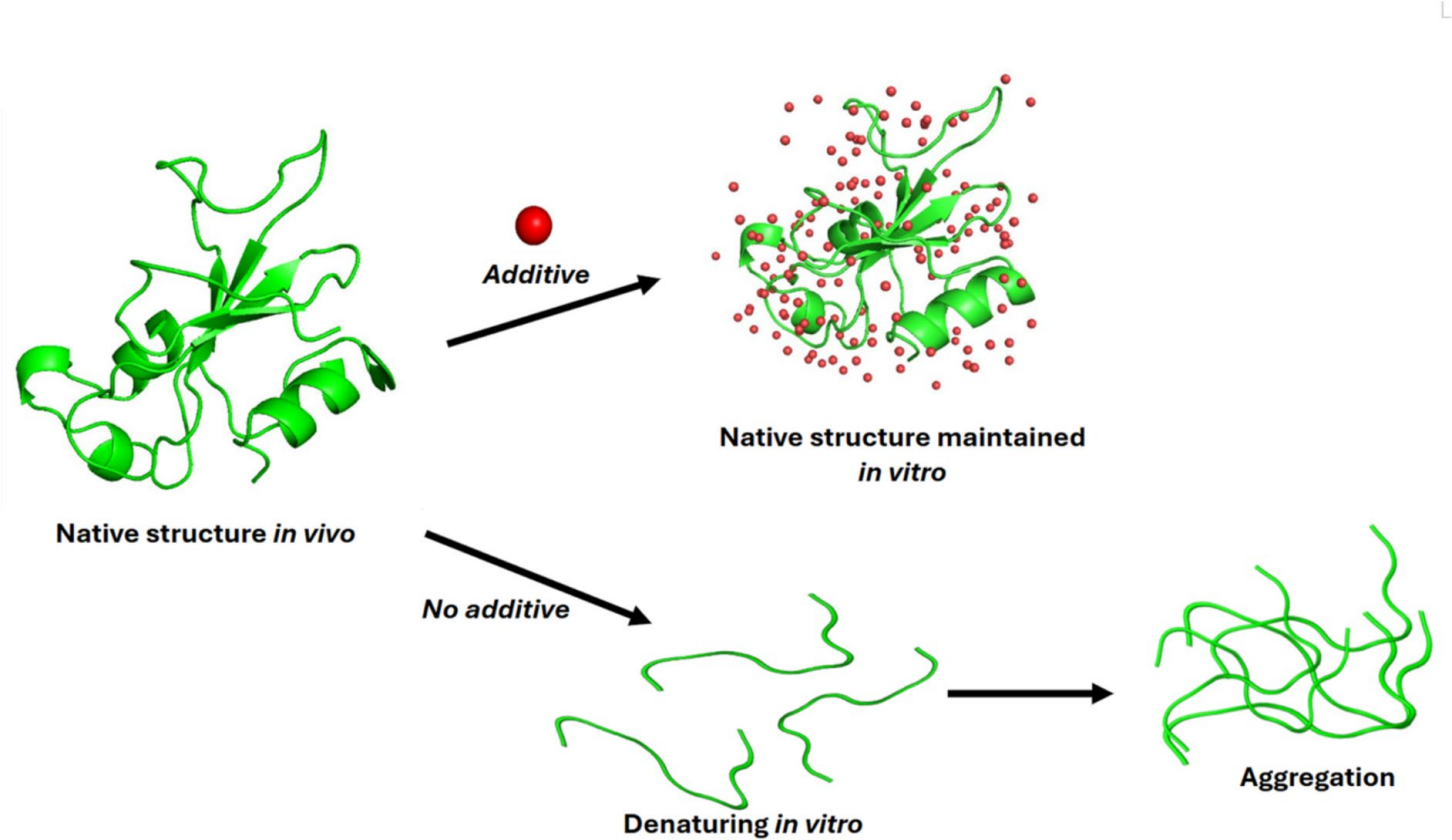
Impact of additive small molecules on the solubility of protein in vitro. A schematic of a protein structure in solution in the presence of additives, leading to a native structure well-maintained, or without additives, leading to denaturation and aggregation of protein

To mitigate the many issues that can affect a purified protein in solution, additives have been used in an attempt to stabilise the protein and keep it as close to its native state as possible. The presence of small molecules may alter the binding of the amino acids to the molecules and thus alter the folding or unfolding events (Jackson 1998), as illustrated in Fig. 1. As protein stability is dependent on the primary amino acid sequence, combined with the environment and its various external factors, this implies that the most favourable conditions to stabilise a protein are sample dependent and should be empirically determined for each protein.

Increasing protein stability also ensures that the protein solubility is improved (Bagby et al. 2001; Golovanov et al. 2004). Globular proteins evolved in water-based solutions and so to minimise entropy; hydrophobic collapse is a driver of cytosolic protein folding into its native structure (Pace et al. 2004). During hydrophobic collapse, the non-polar residues to the amino acids that comprise the protein are buried within the centre to minimise unfavourable interactions with water, forming a hydrophobic core (Dill 1985). This allows the hydrophilic resides on the surface to interact with the polar solution and form a hydration shell around the protein (Petersen et al. 1998). Whilst a soluble protein can be unstable, or a stable protein can be in a non-soluble phase (Bagby et al. 2001), when dealing with downstream biophysical or biochemical characterisation and improving the sample conditions for these, the desired outcome is the same. Protein that is in as close to its native state as possible that stays in solution without any noticeable precipitation or aggregation to add noise to the experiments. Protein solubility is related to protein stability and can be thought of as the concentration of a protein in solution in equilibrium with the solid phase (Kramer et al. 2012). The solubility of a protein is influenced by the protein primary structure, as well as the factors in the aqueous environment such as pH, ionic strength, and temperature. Addition of small molecules can also be a big factor (RiesKautt and Ducruix 1997). As there are large overlaps between the impact small molecules can have on both the stability and solubility of a protein, this review has focused on solubility, but as the preferred outcome is a protein sample that can be used in downstream applications, this review focuses on the outcome of the small molecules rather than the direct mechanisms of how this is achieved.

There are many ways of introducing protein stability and solubility, starting from the overexpression system and the initial cell lysis. We will not address these here but draw the reader’s attention to (Structural Genomics et al. 2008), which highlights some of the pitfalls at the early stages of obtaining a protein sample that is homogenous and stable in solution.

Stability and solubility can often be improved by optimising the pH of the buffer system or by increasing or reducing the salt concentration (Huynh and Partch 2015). It is often beneficial to remove certain components of the purification buffers, such as imidazole, which is chaotropic and may have a destabilising effect on proteins.

A number of studies have been undertaken to identify small molecules that can enhance protein stability and solubility and thereby aid biophysical characterisation of a protein, starting with the addition at protein expression and purification, and continued as an additive throughout the work. Often, the answer is to use substrate or product mimics that already interact with the protein in question, such as the increase found in the melting temperature of the human sulfotransferase 1C1. In the presence of the product PAP (3′-phosphoadenosine-5′-phosphate), a product of the enzymatic reaction of the protein, the Tm increased compared to the protein on its own (Vedadi et al. 2006). However, whilst adding substrate or product to the sample may very well stabilise the sample for structural studies, it will not be suited for biochemical assays where the mechanism is being elucidated, or for biophysical determination of the binding affinities to either. It is therefore better to identify generic small molecules with a propensity for stabilising a protein in solution without affecting the activity or the mechanism.

Various groups have looked at identifying general small molecules that can be used as “magic bullets” to improve a generic protein’s stability (Zacharioudakis and Gavathiotis 2022), and this review is attempting to catalogue some of these efforts. The focus has been on small molecules that are readily available and affordable to any researcher. Working with membrane proteins comes with its own set of challenges as the system is now a protein-detergent micelle or a protein-lipid nano disc, which adds another level of complexity, and we have therefore focused on soluble proteins in this manuscript. We will also discuss the most common ways of determining protein stability whilst screening for small molecules below, with links to appropriate protocols for these studies.

## Common small molecules used to optimise protein stability

### Amino acids

Amino acids have been identified as stabilising agents to improve protein stability for biophysical studies and protein crystallisation. The addition of additives seems to improve solubility for structural studies (Lu et al. 2008), but has also been shown to be key to improve the protein stability for studies in solution (Platts and Falconer 2015). An overview of concentration ranges used for the small molecules listed is presented in Table 1.

**Table 1:** Osmolytes and the concentration ranges that have been reported

Osmolyte	Concentration range reported	References
Arginine	0.1–2 M	(Vagenende et al. 2009), (Platts and Falconer 2015)
Serine	1 M	(Arakawa and Timasheff 1985)
Proline	0.5–1.5 M	(Tanidjaja and Damodaran 2021)
Glutamate/aspartate	0.025–0.1 M	(Golovanov et al. 2004; Lu et al. 2008)
Histidine/imidazole	0.05–0.2 M	(Hamilton et al. 2003)
Sucrose	0.3–1 M	(Arakawa et al. 1993)
Trehalose	0.2–1 M	(Lin and Timasheff 1996)
Glycerol	3–50%	(Back et al. 1979; Meng et al. 2004)
Sorbitol	0.25–2 M	(Xie and Timasheff 1997)
Betaine	0.015–0.5 M	(Caldas et al. 1999)

#### Arginine

L-arginine has been commonly used as a stabiliser of proteins, and more frequently to increase their solubility, and often in combination with other small molecules, such as the protein NaNMAT from *Bacillus anthracis*, which was found to be aggregated when dialysed over night to remove imidazole from the running buffer. The loss of protein due to aggregation was determined to be more than sixfold, according to dynamic light scattering (DLS) measurements. Addition of 50 mM arginine and 50 mM glutamine improved the solubility, and the protein could be purified in quantities suitable for crystallisation trials. Additional screens, using self-interaction chromatography (Tessier et al. 2002; Gabrielsen et al. 2010), allowed the optimisation of the concentration of the amino acids with the addition of a third small molecule (trehalose) to improve the crystallisation conditions further (Lu et al. 2008).

L-arginine has also been used to stabilise BSA at concentrations below 100 mM (Platts and Falconer 2015) and, at 50 mM in combination with 50 mM glutamine, has been shown to reduce aggregation in 6 different proteins and increase the yield up to 8 times (Golovanov et al. 2004), using NMR to screen the conditions.

Non-canonical forms of arginine, such as acetyl-L-arginine, have also been shown to be as effective in reducing aggregation of hen egg-white lysozyme, as well as bovine hemoglobin and γ-globulin, as arginine itself, as determined by light scattering intensities at 400 nm and circular dichroism (CD) (Miyatake et al. 2016). It is worth noting that at higher pH (pH 10 or above), the non-canonical acetyl-L-arginine seem to be better at stopping aggregation than the canonical one. This suggests that the contribution from hydrophobic interactions play a key role in the stabilisation.

**Fig. 2:**
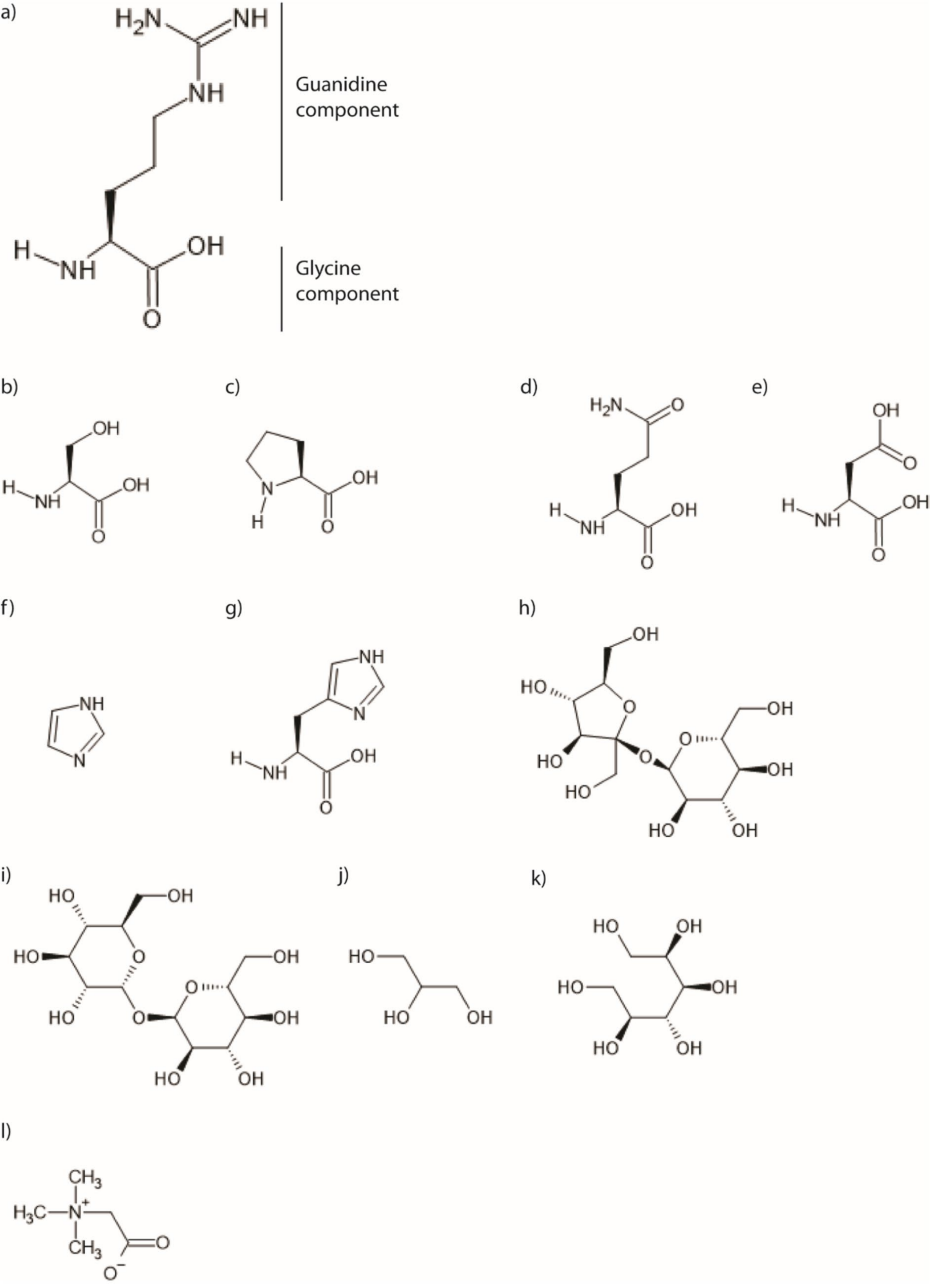
Small molecules used for improving protein stability. Structural representations of the small molecules described in this review. **a** Arginine, with the two parts highlighted as described in Sect. "Arginine". **b** Serine. **c** Proline. **d** Glutamate. **e** Aspartate. **f** Imidazole. **g** Histidine. **h** Sucrose. **i** Trehalose. **j** Glycerol. **k** Sorbitol. **l** Betaine

It has been suggested that arginine works in two distinct ways when stabilising protein, where the guanidine side-chain component is separate from the glycine component (Fig. 2a). Platts and Falconer (2015) explored the contribution of each component by using glycine, guanidine-HCl or arginine in their experiments, and identified that depending on concentration of arginine, the behaviour of the small molecule altered. When below 100 mM, arginine mimicked the effects of glycine upon the three enzymes studied whereas when the concentration of arginine was kept above 100 mM arginine, the protein was becoming destabilised (Kim et al. 2016). Arginine binds specifically to aromatic and charged side chains of amino acids on protein surfaces in solution due to the cation − π interaction and salt-bridge formation (Shukla and Trout 2010).

#### Serine and proline

There is less literature on the effects of serine (Fig. 2b) on protein stabilisation; however, it has been found that it can stabilise lysozyme against thermal denaturation (Arakawa and Timasheff 1985). Serine can act as a protein stabiliser as shown by Trevino et al. (2007) who determined the effect of serine on Ribonuclease A solubility and stability. The ability of serine to improve solubility of a protein appears to be down to the polar properties of the amino acid, which allows it to bind to water, and favourably hydrate the protein of interest (Trevino et al. 2007). Proline (Fig. 2c) is rarely used but has been reported to work as a chaperone when added to solutions when protein is being refolded (Kumar et al. 1998). More recently, Grazi et al. (2025) used proline to stabilise globular proteins such as ubiquitin, using molecular dynamics simulations. Proline appears to bind to the protein of interest mainly through its carboxyl moiety rather than the pyrrolidine ring. Unbound proline can self-associate and thus stabilise proteins by trapping water residues around the protein by a cage effect (Gazi 2025). Both serine and proline have been shown to increase the denaturation temperature of BSA (Tanidjaja and Damodaran 2021) and increase the solubility while reducing the formation of aggregates of cytochrome c and myoglobin (Javanshad and Venter 2021).

#### Glutamate and aspartate

Glutamate (Fig. 2d) was shown to lead to preferential hydration of albumin, lysozyme, lactoglobulin and tubulin and thereby increase protein stability (Ahlgren et al. 2023). Both amino acids (Fig. 2d, e) have been shown to increase the thermal stability of pig-heart malate dehydrogenase (*pmh*-MDH) (Jensen et al. 1996), as well as stabilising RNase A from the pancrease in a DSC assay (Izzi et al. 2024). A mix of the two has also been shown to protect RNAseA from denaturing agents such as urea or guanidinium hydrochloride as well as increased temperatures. This protection is thought to come through a gain of translational entropy of water and co-solute ions, as well as reducing the solvent accessible surface area of the protein (Izzi et al. 2024). Moreover, glutamate has been found to improve solubility of a range of proteins such as of ribonuclease A (Trevino et al. 2007), as well as the near insoluble proteins human MAGO and several WW domains from fruit fly (Shukla and Trout 2010). The two last examples worked particularly well when in combination with arginine, and a synergistic effect was seen at 50 mM of each of the two amino acids (Shukla and Trout 2010). This synergy has also been shown by Golovanov et al. (2004) and Lu et al. (2008). Glutamine has also been used in combination with arginine, and the effect of the two seems to be synergistic (Golovanov et al. 2004; Lu et al. 2008).

#### Histidine/imidazole

Whilst standard advice is to remove imidazole (Fig. 2f) from the purification buffer as soon as possible after IMAC elution, as it often has a stabilising effect on the protein, the opposite effect has also been observed. Recombinantly expressed single-chain Fv antibody fragments have been shown to have limited solubility and stability when stored frozen (Hamilton et al. 2003). The authors noted that the samples did not aggregate when kept in the elution buffer from the Ni^2+^-column, and therefore assumed it was either due to the presence of high NaCl concentration (of 1 M), or the presence of imidazole that kept the protein in solution. Further screening revealed that the presence of at least 50 mM imidazole could maintain the Fv antibody fragment in solution at concentrations of 400 µg/mL, compared with NaCl containing solutions where the protein could only be maintained at 200 µg/mL. The authors observed similar results when using L-Histidine (Fig. 2g), but as imidazole is much cheaper and had the same stabilising effect, they continued with imidazole (Hamilton et al. 2003). Histidine does seem to have a particular use in improving the solubility of antibodies, as well as in reducing aggregations. Interactions between the monoclonal antibody COE3 and histidine have been studied using molecular dynamics simulations (Saurabh et al. 2022), which showed that the interaction is highly dependent on the pH of the solution the protein is maintained in, and histidine tends to bind more readily onto the protein at a neutral pH rather than a high pH where electrostatic repulsion will keep histidine away from the positively charged proteins. Histidine tends to bind to the hydrophobic surface residues of a protein, and in cases of antibody-binding, where these surfaces are often associated with the hinge regions, histidine can impact the protein flexibility (Saurabh et al. 2022). Histidine thus stabilised proteins by covering the hydrophobic regions of native or partially unfolded proteins and hinders unfolding as well as aggregate formation (Saurabh et al. 2022). Shi et al. (2007) has also shown that imidazole has a chaperone-like nature as it promoted eGFP refolding after chemical denaturation.

### Sugars

#### Sucrose

Sucrose (Fig. 2h) stabilises proteins in vitro against chemical and thermal denaturants. It has been long known sucrose stabilises through rendering the unfolded protein conformation less thermodynamically favourable (Lee and Timasheff 1981) and decades later it is still utilised and researched (Shimizu et al. 2017; Jonsson et al. 2024). Sucrose acts as a crowding agent, increasing the volume packing density of the protein by inducing the protein into a conformation that allows the smallest solvent-accessible area (Graziano 2012). The more compact protein conformation allows for increased stability. Sucrose was also found to increase both the melting and aggregation temperatures of the diphtheria toxin CRM197 (McClure et al. 2018) and stabilise rhDNas (Chan et al. 1996), myoglobin (Ahlgren et al. 2023; Jonsson et al. 2024), lysozyme (Jonsson et al. 2024) and bovine serum albumin (BSA) (Tanidjaja and Damodaran 2021) by creating a protective hydration shell around the protein (Ahlgren et al. 2023). Sucrose has also been shown to inhibit dimerisation of IL-1ra (Chang et al. 1996) as well as aggregation of rhIFN-γ (Kendrick et al. 1998) or bFGF (Wang et al. 1996). Moreover, sucrose is also commonly used as a cryoprotectant, for both x-ray crystallography and cry-electron microscopy, where proteins are cooled to 100 K (Pflugrath 2015; Jonsson et al. 2024).

#### Trehalose

Trehalose (Fig. 2i) is another sugar, composed of two glucose molecules which are commonly used to enhance protein stability. Trehalose has been seen to increase the thermal stability of RNase A (Lin and Timasheff 1996) and rhDNase (Chan et al. 1996) and to inhibit aggregation of aFGF (Tsai et al. 1993). Trehalose has also been shown to stabilise firefly luciferase and to reduce aggregation (Rasouli et al. 2011). Ahlgren et al. (2023) compared the stabilising effects of sucrose and trehalose on myoglobin using a range of methods including molecular dynamics simulations and determined that trehalose is the more stabilising agent of the two. Trehalose works in a similar fashion to sucrose, although it is better at mimicking the proteins' natural aqueous environment, due to the rotational motion between the two dihedrals of trehalose being lower than the dihedrals of sucrose, which leads to less perturbation of the protein structure (Ahlgren et al. 2023).

#### Other sugars

Other sugars have also been utilised, such as lactose or mannitol (Chang et al. 1993). The effects on protein stability and homogeneity appear to be depending on their concentration, where a concentration of 5% (or 0.3 M) sugar has been suggested to the lowest concentration required (Arakawa et al. 1993).

### Other osmolytes

#### Glycerol

Glycerol (Fig. 2j) is often used in protein solutions. Vagenende et al. (2009) suggested glycerol prevents protein aggregation through stabilising aggregation-prone protein intermediates through its amphipathic property; its hydrophobic backbone interacting with hydrophobic regions of the aggregate, while the polar hydroxyl groups interact with the polar solvent, similar to the mechanism of stabilisation by arginine.

Glycerol has previously been found to increase the thermal stability and reduce protein aggregation of trypsin (Pazhang et al. 2016), *Bacillus subtilis* lipase A, cellobiohydrolase I from *Trichoderma reesei*, endoglucanase from *Penicillium verruculosum* (Wang et al. 2022) and the diphtheria toxin CRM197 (McClure et al. 2018). Wang et al. (2022) suggest that glycerol drives protein stability by increasing the number of internal hydrogen bonds that hold together the protein and by blocking the sites on the protein surfaces that water can interact with. However, glycerol may bind to the active sites of enzymes, depending on the properties of the residues in the active site and thus inhibit or reduce the enzymatic rate of specific enzymes (Wang et al. 2022).

#### Sorbitol

The sugar alcohol sorbitol (Fig. 2k) has long been used to stabilise proteins. It was found to stabilise both the native and denatured state of ribonuclease A and preferentially bound to the former, which encourages the stable protein form. Sorbitol-based stabilisation of ribonuclease A does seem to be pH dependent, with favoured pH at 2.0 (Xie and Timasheff 1997). Moreover, D-sorbitol was shown to be a stabilising molecule, as it increased the thermostability of trypsin (Pazhang et al. 2016) and CRM197 (McClure et al. 2018).

#### Betaine

Glycine betaine (Fig. 2l) is a trimethylamine that can protect against urea through forming strong hydrogen bonds and electrostatic interactions with the denaturant, reducing the contact it has with the protein (Kumar and Kishore 2016). It also protects against thermal induced denaturing in vitro as identified by Caldas et al. (1999); however, for some proteins, it has destabilising effects at high and low pH values (Singh et al. 2009), so may not be applicable in all buffer conditions. Its concentration must also be considered as betaine has been found to decrease then increase stability of a mini-protein with increasing concentration (Acharyya et al. 2020). Against heat, betaine functions by both destabilising the denatured state of the protein and lowering the free energy of the native state, resulting in preference for the native state conformation. It has unfavourable interactions with the backbone but favourable interactions with the side chains that dominate in the native state (Auton et al. 2011), forming strong cation pi bonds to aromatic groups with its methylated ammonium moiety, similar to the mechanism of arginine (Capp et al. 2009; Rapp et al. 2014). Glycine betaine was also found to stabilise lysozyme and prevent its aggregation (Arya et al. 2024).

## Common methodologies to screen small molecules

It is highly unlikely that there is a “magic bullet” that can solve all problems with protein instability and that the addition of small molecules is dependent on the properties of the particular protein. Having a way to quickly determine the impact of adding small molecules to the sample is therefore useful to identify the best additives for that system. We present a few common methods which use small amounts of sample, and which will yield results quickly. These methods have been commonly used in identifying protein stability and aggregation state. For convenience, we have added links to protocols for the methods offered in our research facility. An overview of commonly used techniques, with advantages and disadvantages, is listed in Table 2.

**Table 2:** Overview of the common methods to assess impact of small molecules on protein sample

Biophysical technique	Sample criteria	Advantages	Disadvantages	Reference
Circular dichroism	0.2–0.8 mg /ml 750 µl	Low volume Label free Inexpensive	Low throughput	https://www.gla.ac.uk/colleges/mvls/shared-research-facilities/protein-analysis/
Differential scanning calorimetry	1 mg/ml protein 1000 µl	Label free Complete thermal dynamic profile Inexpensive	Large sample quantity Low throughput	https://doi.org/10.1016/j.abb.2012.09.008
Differential scanning fluorimetry	10 µg protein per well in 96 well plate	Low volume High throughput Inexpensive	Requires a fluorophore label Limited to soluble proteins Limited to only Tm data	https://www.gla.ac.uk/colleges/mvls/shared-research-facilities/protein-analysis/
Nano DFS	4–10 µl in a capillary	Low volume Label free High throughput Inexpensive	Requires more than one tryptophan Limited to only Tm data	https://doi.org/10.1016/S0006-3495(94)80799-4

### Circular dichroism

A circular dichroism (CD) spectrophotometer measures the difference between the left and right circular polarised light of a chiral sample. When proteins are folded, they have highly asymmetric secondary structural elements, such as α-helices and β-sheets, which have characteristic CD spectra.

**Fig. 3:**
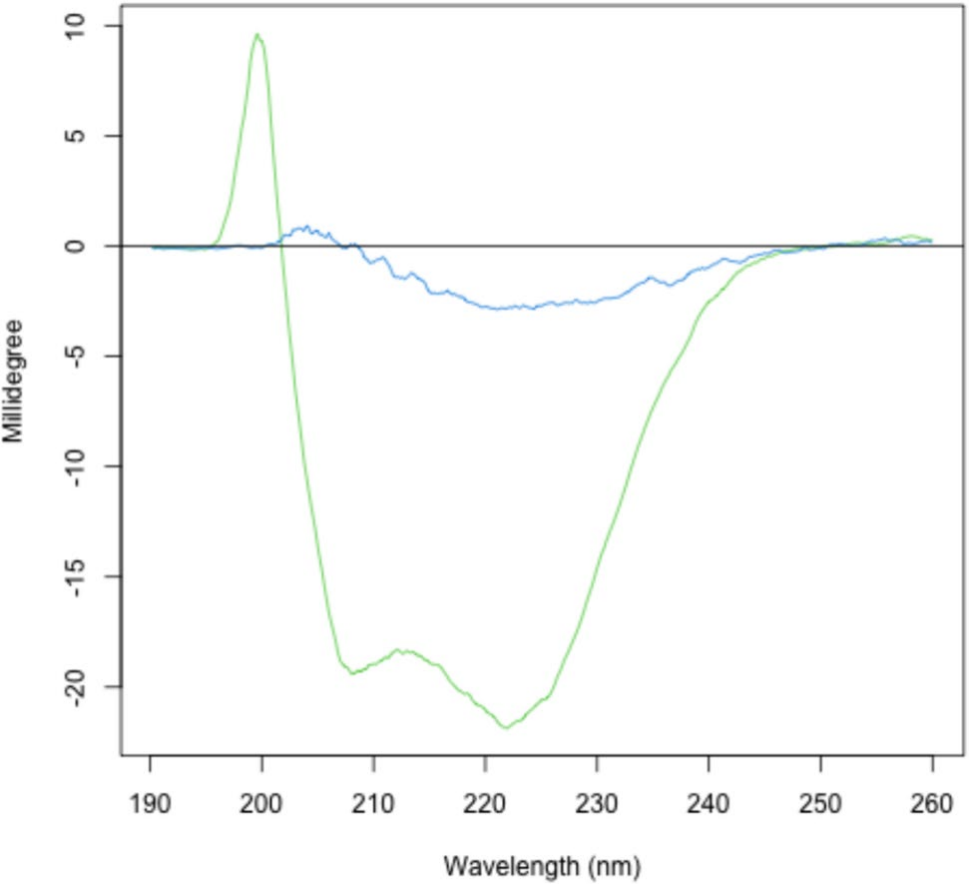
CD spectra of protein sample. CD spectra of protein sample at 15°C (green line) and the same sample at 85°C. Data were collected at the Neil Bulleid Integrated Protein Analysis facility, University of Glasgow on a Jasko J810

The CD spectra in the far UV region (190–240 nm) measure the peptide bond alignment. This can give an estimate on the number of different types of secondary structure elements there are in a protein or peptide. When proteins unfold, they lose these highly ordered structures and the CD spectra changes. With the use of Peltier devices, the temperature inside a cell can be varied and CD studies at increasing temperatures can be used to provide information on the stability of proteins. The thermodynamics of protein unfolding can be investigated by monitoring the ellipticity at a single wavelength and information about the unfolding pathway is greatly increased by collecting a full spectrum as a function of temperature. Unfolding studies are usually preformed at a single point such as 222 nm for α-helical, 218 nm for β-sheets and 225 or 2200 nm for collagen-like proteins. The CD spectra range is set from 260 to 190 nm at regular intervals between 10°C and 85°C and as the temperature rises and the protein unfolds, the CD ellipticity at a single point wavelength and a full CD spectrum at each temperature interval are recorded (Fig. 3). To check if the unfolding is reversible, the CD can run reverse scan intervals from 85°C to 10°C. A protocol describing a basic CD experiment can be found https://www.gla.ac.uk/colleges/mvls/shared-research-facilities/protein-analysis/.

### Differential scanning calorimetry

Differential scanning calorimetry (DSC) is a powerful and versatile technique that can give a complete thermodynamic profile of a protein. DSC is used mainly for the study of thermal transitions in dilute solution, such as protein folding, DNA melting and lipid bilayer transitions. Typical protein concentrations are 0.1–3 mg/ml, with a required sample volume of 0.7–1.8 ml. The temperature range is normally between 5 and 130°C. It measures the energy required to disrupt the interactions stabilising the tertiary structure of a protein. The technique introduces heat simultaneously to a protein sample in a cell and to the protein buffer in a reference cell. As the protein is heated (the sample absorbs heat relative to the reference cell) and unfolds, an endothermic event is represented on the DSC thermogram by a negative peak, meaning the heat flow into the sample is less than that into the reference). The area under the endothermic peak is proportional to the enthalpy change (ΔH) of the protein unfolding. Analysis of the DSC trace provides the melting temperature (Tm) and enthalpy of unfolding of the protein (Kuril 2024).

### Extrinsic fluorescence measurements

Differential scanning fluorimetry (DSF) is a biophysical technique that has been used for many years to detect unfolding and folding states of proteins, and is otherwise known as a thermal shift assay (Gao et al. 2020). It provides a reliable method to measure protein unfolding in a controlled thermal setting by measuring fluorescence emission form a fluorescent dye as the temperature increases. In DSF, the fluorescence intensity is plotted as a function of temperature; this generates a sigmoidal curve that can be used to work out the Tm of the protein. DSF is an excellent tool for scouting the optimal buffer composition for a protein, identifying the ideal buffer base, pH and ionic strength as well as small molecules to enhance protein stability(Huynh and Partch 2015).

**Fig. 4:**
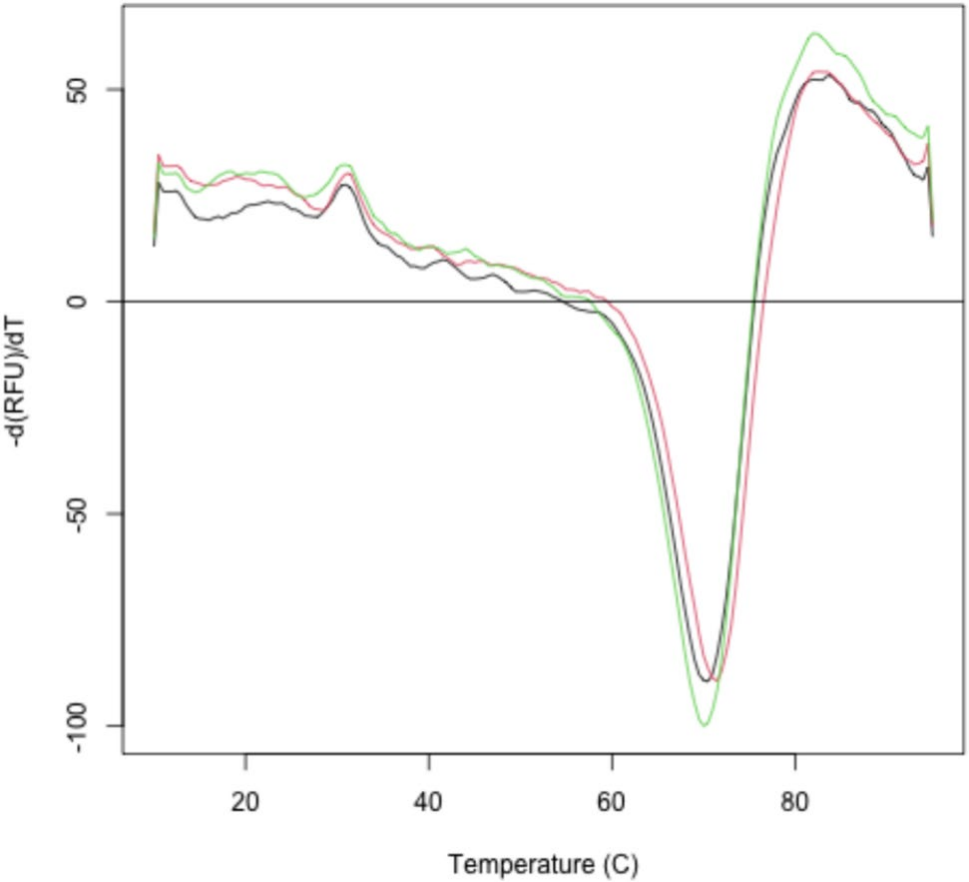
Differential scanning fluorimetry data. Data of protein sample in triplicate (red, green and black) using SYPRO-Orange as the dye. Data were collected at the Neil Bulleid Integrated Protein Analysis facility at the University of Glasgow., on a BioRad RT-PCR thermal cycler

Fluorescent dyes, when in aqueous solutions, are quenched, but when bound to hydrophobic parts of a protein as it unfolds will fluoresce and emit a signal. Various commercial dyes such as bi-ANS, Nile red and SYPRO orange are suitable for measuring protein folding. The protein, dye and buffer are mixed in a well of 96 well plate and placed in a real time polymerase chain reaction. As the temperature increases, the protein unfolds exposing the hydrophobic interior which the fluorophore dye attaches to resulting in an increase in fluorescence. When the unfolded protein starts to aggregate, the dye is displaced and the fluorescence decreases (Fig. 4). For convenience, a protocol routinely used in our research facility can be found here: https://www.gla.ac.uk/colleges/mvls/shared-research-facilities/protein-analysis/.

### Intrinsic fluorescence measurements

An alternative to the standard DSF experiment is nano-differential scanning fluorimetry. This method relies on the dominant intrinsic fluorophore of a protein caused by the side chain of the amino acid tryptophan, characterised by the indole moiety which has an excitation wavelength near 280 nm. The emission wavelength is sensitive to the environment, and in a polar environment, e.g. aqueous solution or at the hydrophilic exterior of the protein, the emission wavelength is 350 nm. In a non-polar environment, organic solvent or in the hydrophobic core of a protein, the emission wavelength is 330 nm. Label-free DSF was developed in 2014 and is marketed as nano-DSF. This technique measures the change of intrinsic tryptophan residues at 330 nm and 350 nm. As the protein unfolds, the fluorescence intensity ratio of 350 nm to 330 nm changes, and the T_m_ can be extrapolated (https://nanotempertech.com/nanodsf/). This method can be used when the protein is not a suitable candidate for mixing with a fluorescent dye, e.g. membrane proteins, a membrane associated protein, or globular proteins with exterior hydrophobic pockets, e.g. BSA. Alexandrov et al. (2008) describe the use of nano–DSF and microscale thermophoresis (MST) to measure the differences in thermal stability of soluble and membrane proteins with the addition of small molecules and screening with different detergents. This method has the advantages of using a small amount of protein with as little as 10 µg per test, with a quick data output.

### Other biophysical techniques

The Prometheus Panta from Nanotemper (https://nanotempertech.com/prometheus/biologics/) allows the use of four technologies in one read-out; nano-DFS, backreflection, dynamic (DLS) and static light scattering (SLS) to give a thermal stability profile with information on aggregation and purity. The system monitors both the intrinsic fluorescence and scattering properties in one experiment, and the data for these technologies are collected along a thermal ramp 15–95°C at a rate of 0.1–7°C/min. Up to 24 capillaries with small volumes of 10 µl of sample can be measured in one experiment. The readout from these four technologies can not only determine the Tm, but also quantify the turbidity, the onset temperature of aggregation, and the Polydispersity index, which gives a distribution of the populations in the sample based on size.

There are a range of other methods that can be used to determine the effect of a small molecule on a protein target, such as small-angle x-ray scattering (SAXS) or nuclear magnetic resonance (NMR). SAXS is a method which will determine the radius of gyration (Rg), or how globular or elongated the protein is. Changes in the Rg can be monitored as small molecules are added to the conditions. Unless there is an available in-house SAXS source, the samples require shipping to a synchrotron facility (such as beamline B21 at Diamond Light Source, UK) for data collection, which can make this a slower process. NMR of a protein sample will give H-spectra which will show if the protein is ordered or disordered. Provided the equipment is available on site, it can be a relatively fast method for initial screening. However, if the improved stability is not dramatic, it can be difficult to quickly quantify which condition is to be preferred without further experiments and potential labelling of the protein.

## Conclusions

Small molecules can enhance the thermal stability and solubility of a protein and make a big difference to a protein’s stability and tendency to aggregate. Addition of an appropriate small molecule may rescue an experiment which was not possible without the component. We have attempted to highlight some of the more commonly used small molecules, with a focus on those which are readily available and affordable, as they may have to be present throughout purification and down-stream experiments. As the combination of small molecule and protein needs to be determined empirically, we have presented a few methods to monitor the impact of the small molecule and provided links to our current protocols. It is important to recognise that the impact of small molecules is dependent on the protein and the environment the protein is in, and that they may have unintended consequences in any downstream applications (such as adding free amine groups to samples that will later be cross-linked using ethanolamide linking). The researcher should understand their system and the intended use of their samples before using additives to stabilise their protein.

## Data Availability

No datasets were generated or analysed during the current study.

## References

[CR1] Acharyya A, Shin D, Troxler T, Gai F (2020) Can glycine betaine denature proteins? Phys Chem Chem Phys 22(15):7794–7802. 10.1039/D0CP00397B 32242578 10.1039/d0cp00397b

[CR2] Ahlgren K, Olsson C, Ermilova I, Swenson J (2023) New insights into the protein stabilizing effects of trehalose by comparing with sucrose. Phys Chem Chem Phys 25(32):21215–21226. 10.1039/D3CP02639F 10.1039/d3cp02639f 37534799

[CR3] Alexandrov AI, Mileni M, Chien EYT, Hanson MA, Stevens RC (2008) Microscale fluorescent thermal stability assay for membrane proteins. Structure 16(3):351–359. 10.1016/j.str.2008.02.004 18334210 10.1016/j.str.2008.02.004

[CR4] Arakawa T, Timasheff SN (1985) The stabilization of proteins by osmolytes. Biophys J 47(3):411–414. 10.1016/S0006-3495(85)83932-1 3978211 10.1016/S0006-3495(85)83932-1 PMC1435219

[CR5] Arakawa T, Prestrelski SJ, Kenney WC, Carpenter JF (1993) Factors affecting short-term and long-term stabilities of proteins. Adv Drug Deliv Rev 10(1):1–28. 10.1016/s0169-409x(00)00144-7 10.1016/s0169-409x(00)00144-7 11259845

[CR6] Arya A, Rathee J, Kishore N (2024) Analyzing synergy in combined influence of sorbitol and glycine betaine on protein stability: thermodynamic and mechanistic insights. J Mol Liq 400:124515. 10.1016/j.molliq.2024.124515

[CR7] Auton M, Rosgen J, Sinev M, Holthauzen LM, Bolen DW (2011) Osmolyte effects on protein stability and solubility: a balancing act between backbone and side-chains. Biophys Chem 159(1):90–99. 10.1016/j.bpc.2011.05.012 21683504 10.1016/j.bpc.2011.05.012 PMC3166983

[CR8] Back JF, Oakenfull D, Smith MB (1979) Increased thermal stability of proteins in the presence of sugars and polyols. Biochemistry 18(23):5191–5196. 10.1021/bi00590a025 497177 10.1021/bi00590a025

[CR9] Bagby S, Tong KI, Ikura M (2001) Optimization of protein solubility and stability for protein nuclear magnetic resonance. Methods Enzymol 339:20–41. 10.1016/s0076-6879(01)39307-2 11462812 10.1016/s0076-6879(01)39307-2

[CR10] Caldas T, Demont-Caulet N, Ghazi A, Richarme G (1999) Thermoprotection by glycine betaine and choline. Microbiology (Reading) 145(Pt 9):2543–2548. 10.1099/00221287-145-9-2543 10517607 10.1099/00221287-145-9-2543

[CR11] Capp MW, Pegram LM, Saecker RM, Kratz M, Riccardi D, Wendorff T, Cannon JG, Record MT Jr (2009) Interactions of the osmolyte glycine betaine with molecular surfaces in water: thermodynamics, structural interpretation, and prediction of m-values. Biochemistry 48(43):10372–10379. 10.1021/bi901273r 10.1021/bi901273r PMC2783871 19757837

[CR12] Chan HK, Au-Yeung KL, Gonda I (1996) Effects of additives on heat denaturation of rhDNase in solutions. Pharm Res 13(5):756–761. 10.1023/a:1016007818575 8860433 10.1023/a:1016007818575

[CR13] Chang BS, Randall CS, Lee YS (1993) Stabilization of lyophilized porcine pancreatic elastase. Pharm Res 10(10):1478–1483. 10.1023/a:1018979410338 7505940 10.1023/a:1018979410338

[CR14] Chang BS, Beauvais RM, Arakawa T, Narhi LO, Dong A, Aparisio DI, Carpenter JF (1996) Formation of an active dimer during storage of interleukin-1 receptor antagonist in aqueous solution. Biophys J 71(6):3399–3406. 10.1016/S0006-3495(96)79534-6 8968609 10.1016/S0006-3495(96)79534-6 PMC1233827

[CR15] Dill KA (1985) Theory for the folding and stability of globular proteins. Biochemistry 24(6):1501–1509. 10.1021/bi00327a032 3986190 10.1021/bi00327a032

[CR16] Gabrielsen M, Nagy LA, DeLucas LJ, Cogdell RJ (2010) Self-interaction chromatography as a tool for optimizing conditions for membrane protein crystallization. Acta Crystallogr D Biol Crystallogr 66(Pt 1):44–50. 10.1107/S0907444909043972 20057048 10.1107/S0907444909043972

[CR17] Gao K, Oerlemans R, Groves MR (2020) Theory and applications of differential scanning fluorimetry in early-stage drug discovery. Biophys Rev 12(1):85–104. 10.1007/s12551-020-00619-2 32006251 10.1007/s12551-020-00619-2 PMC7040159

[CR18] Gazi R, Kumar S, Jana M (2025) Proline concentration driven thermostability and hydration properties of ubiquitin. J Mol Liq 424:127108. 10.1016/j.molliq.2025.127108

[CR19] Golovanov AP, Hautbergue GM, Wilson SA, Lian LY (2004) A simple method for improving protein solubility and long-term stability. J Am Chem Soc 126(29):8933–8939. 10.1021/ja049297h 15264823 10.1021/ja049297h

[CR20] Graziano G (2012) How does sucrose stabilize the native state of globular proteins? Int J Biol Macromol 50(1):230–235. 10.1016/j.ijbiomac.2011.10.025 22085755 10.1016/j.ijbiomac.2011.10.025

[CR21] Hamilton S, Odili J, Pacifico MD, Wilson GD, Kupsch JM (2003) Effect of imidazole on the solubility of a his-tagged antibody fragment. Hybrid Hybridomics 22(6):347–355. 10.1089/153685903771797048 14683594 10.1089/153685903771797048

[CR22] Huynh K, Partch CL (2015) Analysis of protein stability and ligand interactions by thermal shift assay. Curr Protoc Protein Sci 79(1):28.29.21-28.29.14. 10.1002/0471140864.ps2809s79 10.1002/0471140864.ps2809s79 PMC4332540 25640896

[CR23] Izzi G, Campanile M, Del Vecchio P, Graziano G (2024) On the stabilizing effect of aspartate and glutamate and its counteraction by common denaturants. Int J Mol Sci 25(17). 10.3390/ijms25179360 10.3390/ijms25179360 PMC11395698 39273310

[CR24] Jackson SE (1998) How do small single-domain proteins fold? Fold des 3(4):R81–R91. 10.1016/S1359-0278(98)00033-9 9710577 10.1016/S1359-0278(98)00033-9

[CR25] Javanshad R, Venter AR (2021) Effects of amino acid additives on protein solubility-insights from desorption and direct electrospray ionization mass spectrometry. Analyst 146(21):6592–6604. 10.1039/d1an01392k 34586125 10.1039/d1an01392k

[CR26] Jensen WA, Armstrong JM, De Giorgio J, Hearn MT (1996) Stability studies on pig heart mitochondrial malate dehydrogenase: the effect of salts and amino acids. Biochim Biophys Acta 1296(1):23–34. 10.1016/0167-4838(96)00049-0 8765225 10.1016/0167-4838(96)00049-0

[CR27] Jonsson O, Lundell A, Rosell J, You S, Ahlgren K, Swenson J (2024) Comparison of sucrose and trehalose for protein stabilization using differential scanning calorimetry. J Phys Chem B 128(20):4922–4930. 10.1021/acs.jpcb.4c00022 38733344 10.1021/acs.jpcb.4c00022 PMC11129304

[CR28] Kendrick BS, Carpenter JF, Cleland JL, Randolph TW (1998) A transient expansion of the native state precedes aggregation of recombinant human interferon-gamma. Proc Natl Acad Sci U S A 95(24):14142–14146. 10.1073/pnas.95.24.14142 9826667 10.1073/pnas.95.24.14142 PMC24340

[CR29] Kim NA, Hada S, Thapa R, Jeong SH (2016) Arginine as a protein stabilizer and destabilizer in liquid formulations. Int J Pharm 513(1–2):26–37. 10.1016/j.ijpharm.2016.09.003 27596112 10.1016/j.ijpharm.2016.09.003

[CR30] Kramer RM, Shende VR, Motl N, Pace CN, Scholtz JM (2012) Toward a molecular understanding of protein solubility: increased negative surface charge correlates with increased solubility. Biophys J 102(8):1907–1915. 10.1016/j.bpj.2012.01.060 22768947 10.1016/j.bpj.2012.01.060 PMC3328702

[CR31] Kumar N, Kishore N (2016) Effect of glycine betaine on the hydrophobic interactions in the presence of denaturant: a molecular dynamics study. J Mol Liq 215:104–109. 10.1016/J.MOLLIQ.2015.12.037

[CR32] Kumar TKS, Samuel D, Jayaraman G, Srimathi T, Yu C (1998) The role of proline in the prevention of aggregation during protein folding. Biochem Mol Biol Int 46(3):509–517. 10.1080/15216549800204032 9818090 10.1080/15216549800204032

[CR33] Kuril AK (2024) Differential scanning calorimetry: a powerful and versatile tool for analyzing proteins and peptides. Journal of Pharmaceutical Research International 36(7):179–187. 10.9734/jpri/2024/v36i77549

[CR34] Lee JC, Timasheff SN (1981) The stabilization of proteins by sucrose. J Biol Chem 256(14):7193–7201. 10.1016/S0021-9258(19)68947-7 7251592

[CR35] Lin TY, Timasheff SN (1996) On the role of surface tension in the stabilization of globular proteins. Protein Sci 5(2):372–381. 10.1002/pro.5560050222 8745416 10.1002/pro.5560050222 PMC2143343

[CR36] Lu S, Smith CD, Yang Z, Pruett PS, Nagy L, McCombs D, Delucas LJ, Brouillette WJ, Brouillette CG (2008) Structure of nicotinic acid mononucleotide adenylyltransferase from * Bacillus anthracis *. Acta Crystallogr Sect F Struct Biol Cryst Commun 64(Pt 10):893–898. 10.1107/S1744309108029102 18931430 10.1107/S1744309108029102 PMC2564882

[CR37] McClure SM, Ahl PL, Blue JT (2018) High throughput differential scanning fluorimetry (DSF) formulation screening with complementary dyes to assess protein unfolding and aggregation in presence of surfactants. Pharm Res 35(4):81. 10.1007/s11095-018-2361-1 29508082 10.1007/s11095-018-2361-1

[CR38] Meng FG, Hong YK, He HW, Lyubarev AE, Kurganov BI, Yan YB, Zhou HM (2004) Osmophobic effect of glycerol on irreversible thermal denaturation of rabbit creatine kinase. Biophys J 87(4):2247–2254. 10.1529/biophysj.104.044784 15454427 10.1529/biophysj.104.044784 PMC1304650

[CR39] Miyatake T, Yoshizawa S, Arakawa T, Shiraki K (2016) Charge state of arginine as an additive on heat-induced protein aggregation. Int J Biol Macromol 87:563–569. 10.1016/j.ijbiomac.2016.03.015 26987431 10.1016/j.ijbiomac.2016.03.015

[CR40] Pace CN, Treviño S, Prabhakaran E, Scholtz JM (2004) Protein structure, stability and solubility in water and other solvents. Philosophical Transactions of the Royal Society of London Series B-Biological Sciences 359(1448):1225–1234. 10.1098/rstb.2004.1500 15306378 10.1098/rstb.2004.1500 PMC1693406

[CR41] Pace CN, Grimsley GR, Scholtz JM, Shaw KL (2014) Protein stability. In: eLS. John Wiley & Sons, Ltd (Ed.). 10.1002/9780470015902.a0003002.pub3

[CR42] Pazhang M, Mehrnejad F, Pazhang Y, Falahati H, Chaparzadeh N (2016) Effect of sorbitol and glycerol on the stability of trypsin and difference between their stabilization effects in the various solvents. Biotechnol Appl Biochem 63(2):206–213. 10.1002/bab.1366 25757511 10.1002/bab.1366

[CR43] Petersen SB, Jonson PH, Fojan P, Petersen EI, Petersen MT, Hansen S, Ishak RJ, Hough E (1998) Protein engineering the surface of enzymes. J Biotechnol 66(1):11–26. 10.1016/s0168-1656(98)00153-9 9866858 10.1016/s0168-1656(98)00153-9

[CR44] Pflugrath JW (2015) Practical macromolecular cryocrystallography. Acta Crystallogr F Struct Biol Commun 71(Pt 6):622–642. 10.1107/s2053230x15008304 26057787 10.1107/S2053230X15008304 PMC4461322

[CR45] Platts L, Falconer RJ (2015) Controlling protein stability: mechanisms revealed using formulations of arginine, glycine and guanidinium HCl with three globular proteins. Int J Pharm 486(1–2):131–135. 10.1016/j.ijpharm.2015.03.051 25818064 10.1016/j.ijpharm.2015.03.051

[CR46] Rahban M, Ahmad F, Piatyszek MA, Haertle T, Saso L, Saboury AA (2023) Stabilization challenges and aggregation in protein-based therapeutics in the pharmaceutical industry. RSC Adv 13(51):35947–35963. 10.1039/D3RA06476J 38090079 10.1039/d3ra06476j PMC10711991

[CR47] Rapp C, Goldberger E, Tishbi N, Kirshenbaum R (2014) Cation-pi interactions of methylated ammonium ions: a quantum mechanical study. Proteins 82(7):1494–1502. 10.1002/prot.24519 24464782 10.1002/prot.24519 PMC4057355

[CR48] Rasouli S, Hosseinkhani S, Yaghmaei P, Ebrahim-Habibi A (2011) Effects of sucrose and trehalose on stability, kinetic properties, and thermal aggregation of firefly luciferase. Appl Biochem Biotechnol 165(2):572–582. 10.1007/s12010-011-9276-1 21617898 10.1007/s12010-011-9276-1

[CR49] RiesKautt M, Ducruix A (1997) Inferences drawn from physicochemical studies of crystallogenesis and precrystalline state. Macromolecular Crystallography, Pt A 276:23–59. 10.1016/s0076-6879(97)76049-x 10.1016/S0076-6879(97)76049-X 27799097

[CR50] Saurabh S, Kalonia C, Li Z, Hollowell P, Waigh T, Li P, Webster J, Seddon JM, Lu JR, Bresme F (2022) Understanding the stabilizing effect of histidine on mAb aggregation: a molecular dynamics study. Mol Pharm 19(9):3288–3303. 10.1021/acs.molpharmaceut.2c00453 35946408 10.1021/acs.molpharmaceut.2c00453 PMC9449975

[CR51] Shi R, Pan Q, Guan Y, Hua Z, Huang Y, Zhao M, Li Y (2007) Imidazole as a catalyst for in vitro refolding of enhanced green fluorescent protein. Arch Biochem Biophys 459(1):122–128. 10.1016/j.abb.2006.11.002 17169325 10.1016/j.abb.2006.11.002

[CR52] Shimizu T, Korehisa T, Imanaka H, Ishida N, Imamura K (2017) Characteristics of proteinaceous additives in stabilizing enzymes during freeze-thawing and -drying. Biosci Biotechnol Biochem 81(4):687–697. 10.1080/09168451.2016.1274637 28067593 10.1080/09168451.2016.1274637

[CR53] Shukla D, Trout BL (2010) Interaction of arginine with proteins and the mechanism by which it inhibits aggregation. J Phys Chem B 114(42):13426–13438. 10.1021/jp108399g 20925358 10.1021/jp108399g

[CR54] Singh LR, Dar TA, Rahman S, Jamal S, Ahmad F (2009) Glycine betaine may have opposite effects on protein stability at high and low pH values. Biochim Biophys Acta 1794(6):929–935. 10.1016/j.bbapap.2009.02.005 19254782 10.1016/j.bbapap.2009.02.005

[CR55] Structural Genomics C, China structural genomics C, Northeast Structural Genomics C, Graslund S, Nordlund P, Weigelt J, Hallberg BM, Bray J, Gileadi O, Knapp S, Oppermann U, Arrowsmith C, Hui R, Ming J, dhe-Paganon S, Park HW, Savchenko A, Yee A, Edwards A, Vincentelli R, Cambillau C, Kim R, Kim SH, Rao Z, Shi Y, Terwilliger TC, Kim CY, Hung LW, Waldo GS, Peleg Y, Albeck S, Unger T, Dym O, Prilusky J, Sussman JL, Stevens RC, Lesley SA, Wilson IA, Joachimiak A, Collart F, Dementieva I, Donnelly MI, Eschenfeldt WH, Kim Y, Stols L, Wu R, Zhou M, Burley SK, Emtage JS, Sauder JM, Thompson D, Bain K, Luz J, Gheyi T, Zhang F, Atwell S, Almo SC, Bonanno JB, Fiser A, Swaminathan S, Studier FW, Chance MR, Sali A, Acton TB, Xiao R, Zhao L, Ma LC, Hunt JF, Tong L, Cunningham K, Inouye M, Anderson S, Janjua H, Shastry R, Ho CK, Wang D, Wang H, Jiang M, Montelione GT, Stuart DI, Owens RJ, Daenke S, Schutz A, Heinemann U, Yokoyama S, Bussow K, Gunsalus KC (2008) Protein production and purification. Nat Methods 5(2): 135–146. 10.1038/nmeth.f.202 10.1038/nmeth.f.202 PMC3178102 18235434

[CR56] Tanidjaja I, Damodaran S (2021) Influence of amino acids on thermal stability and heat-set gelation of bovine serum albumin. Food Chem 337. 10.1016/j.foodchem.2020.127670 10.1016/j.foodchem.2020.127670 32799159

[CR57] Tessier PM, Lenhoff AM, Sandler SI (2002) Rapid measurement of protein osmotic second virial coefficients by self-interaction chromatography. Biophys J 82(3):1620–1631. 10.1016/S0006-3495(02)75513-6 11867474 10.1016/S0006-3495(02)75513-6 PMC1301960

[CR58] Trevino SR, Scholtz JM, Pace CN (2007) Amino acid contribution to protein solubility: Asp, Glu, and Ser contribute more favorably than the other hydrophilic amino acids in RNase Sa. J Mol Biol 366(2):449–460. 10.1016/j.jmb.2006.10.026 17174328 10.1016/j.jmb.2006.10.026 PMC2771383

[CR59] Tsai PK, Volkin DB, Dabora JM, Thompson KC, Bruner MW, Gress JO, Matuszewska B, Keogan M, Bondi JV, Middaugh CR (1993) Formulation design of acidic fibroblast growth factor. Pharm Res 10(5):649–659. 10.1023/a:1018939228201 7686672 10.1023/a:1018939228201

[CR60] Vagenende V, Yap MG, Trout BL (2009) Mechanisms of protein stabilization and prevention of protein aggregation by glycerol. Biochemistry 48(46):11084–11096. 10.1021/bi900649t 19817484 10.1021/bi900649t

[CR61] Vedadi M, Niesen FH, Allali-Hassani A, Fedorov OY, Finerty PJ, Wasney GA, Yeung R, Arrowsmith C, Ball LJ, Berglund H, Hui R, Marsden BD, Nordlund P, Sundstrom M, Weigelt J, Edwards AM (2006) Chemical screening methods to identify ligands that promote protein stability, protein crystallization, and structure determination. Proc Natl Acad Sci 103(43):15835–15840. 10.1073/pnas.0605224103 17035505 10.1073/pnas.0605224103 PMC1595307

[CR62] Wang YJ, Shahrokh Z, Vemuri S, Eberlein G, Beylin I, Busch M (1996) Characterization, stability, and formulations of basic fibroblast growth factor. Pharm Biotechnol 9:141–180. 10.1007/0-306-47452-2_2 8914191 10.1007/0-306-47452-2_2

[CR63] Wang MH, Sheng YJ, Cui HY, Li AN, Li XJ, Huang H (2022) The role of glycerol in preserving proteins needs to be reconsidered. ACS Sustainable Chem Eng 10(46):15175–15185. 10.1021/acssuschemeng.2c04695

[CR64] Xie G, Timasheff SN (1997) Mechanism of the stabilization of ribonuclease A by sorbitol: preferential hydration is greater for the denatured then for the native protein. Protein Sci 6(1):211–221. 10.1002/pro.5560060123 9007993 10.1002/pro.5560060123 PMC2143517

[CR65] Zacharioudakis E, Gavathiotis E (2022) Targeting protein conformations with small molecules to control protein complexes. Trends Biochem Sci 47(12):1023–1037. 10.1016/j.tibs.2022.07.002 35985943 10.1016/j.tibs.2022.07.002 PMC9669135

